# An Intelligent Proposed Model for Task Offloading in Fog-Cloud Collaboration Using Logistics Regression

**DOI:** 10.1155/2022/3606068

**Published:** 2022-01-25

**Authors:** Muhammad Mazhar Bukhari, Taher M. Ghazal, Sagheer Abbas, M. A. Khan, Umer Farooq, Hasan Wahbah, Munir Ahmad, Khan Muhammad Adnan

**Affiliations:** ^1^Department of Computer Science, National College of Business Administration and Economics, Lahore 54660, Pakistan; ^2^Center for Cyber Security Faculty of Information Science and Technology, Universiti Kebangsaan Malaysia (UKM), Bangi 43600, Selangor, Malaysia; ^3^School of Information Technology Skyline University College, University City Sharjah, Sharjah 1797, UAE; ^4^Riphah School of Computing & Innovation Faculty of Computing, Riphah International University Lahore Campus, Lahore 54000, Pakistan; ^5^Department of Computer Science, Lahore Garrison University, Lahore 54000, Pakistan; ^6^College of Computer Information Technology, American University in Emirates, Dubai, UAE; ^7^Pattern Recognition and Machine Learning Lab Department of Software, Gachon University, Seongnam 13557, Republic of Korea

## Abstract

Smart applications and intelligent systems are being developed that are self-reliant, adaptive, and knowledge-based in nature. Emergency and disaster management, aerospace, healthcare, IoT, and mobile applications, among them, revolutionize the world of computing. Applications with a large number of growing devices have transformed the current design of centralized cloud impractical. Despite the use of 5G technology, delay-sensitive applications and cloud cannot go parallel due to exceeding threshold values of certain parameters like latency, bandwidth, response time, etc. Middleware proves to be a better solution to cope up with these issues while satisfying the high requirements task offloading standards. Fog computing is recommended middleware in this research article in view of the fact that it provides the services to the edge of the network; delay-sensitive applications can be entertained effectively. On the contrary, fog nodes contain a limited set of resources that may not process all tasks, especially of computation-intensive applications. Additionally, fog is not the replacement of the cloud, rather supplement to the cloud, both behave like counterparts and offer their services correspondingly to compliance the task needs but fog computing has relatively closer proximity to the devices comparatively cloud. The problem arises when a decision needs to take what is to be offloaded: data, computation, or application, and more specifically where to offload: either fog or cloud and how much to offload. Fog-cloud collaboration is stochastic in terms of task-related attributes like task size, duration, arrival rate, and required resources. Dynamic task offloading becomes crucial in order to utilize the resources at fog and cloud to improve QoS. Since this formation of task offloading policy is a bit complex in nature, this problem is addressed in the research article and proposes an intelligent task offloading model. Simulation results demonstrate the authenticity of the proposed logistic regression model acquiring 86% accuracy compared to other algorithms and confidence in the predictive task offloading policy by making sure process consistency and reliability.

## 1. Introduction

Information and Communication Technology (ICT) plays a vital role in developing and providing services to several emerging and contemporary applications like virtual and augmented reality, smart grid and energy saving, and wearable cognitive assistance applications [1]. Since these applications need such devices (virtual glasses, fitness bands, and Global Positioning System (GPS) tracking belts, etc.) that require the energy/power to operate, processing capability, and storage capacity too and to execute within a given timeframe. Most of these applications are delay-sensitive; they need real-time data processing and require results in a very short time [2]. The devices, for example, a robot, having multiple sensors contain a low battery with light-weight processing capabilities; often, in most cases, they cannot run for a long period of time or may not make useful decisions in complex situations due to these limitations. Proper utilization of the Internet of Things (IoT) can help to overcome the limitations and drawbacks of these devices. IoT enables things to see and perceive their environment, make collaborative decisions, and make corresponding tasks based on the observed data. Normally, the real-time position of robots can be tracked and a system administrator can send the control commands in case of complex situations. Some computation-related tasks such as complex navigation, natural language processing, and machine learning-based intelligence applications can be forwarded to remote cloud servers to facilitate these IoT devices for performing smooth operations [3]. It may be achieved by a proxy server that is responsible for collecting data from these devices, applying some desired processing, and forwarding it to cloud servers. Cloud Computing, as an on-demand availability of computer system resources, proves to be a better solution for providing ICT services anywhere all the time at a relatively low cost on a pay-per-use basis. It has been providing its services in respect of introducing a unique transformation of conventional applications for more than one decade. It offers not only computing (hardware, software, network services, etc.) resources but the structured models too that are suitable for the range of mid-level to enterprise-level organizations throughout the world [4].

### 1.1. Cloud and IoTs

**Figure 1:**
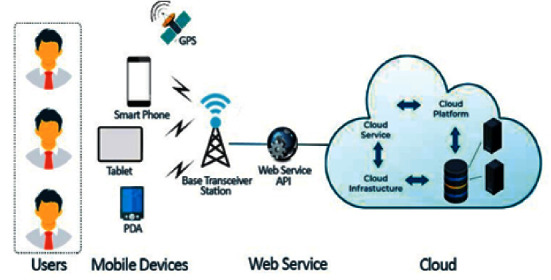
IoT-cloud architecture.

With the advent of the Internet of Things (IoT) and mobile devices, an enormous amount of data is being generated and ever increased day by day. It is expected that these devices would be increased in numbers near about 22 billion and economically ranges from 3.9 trillion dollars to 11.1 trillion dollars per annum till 2025 [5]. Mobile traffic for a smartphone will be expected to be 25 GB on monthly basis and 95% of data will be generated by mobile networks; the mobile data flow rate is exceeding the data flow of static devices by a definite gap, i.e., 66% [6]. These devices have good sensing abilities when communicating and collaborating with other technologies that produce intelligent outcomes to deliver more values to the users. They require a prompt response and immediate action from a remote cloud data center as a cloud possesses a resource-rich environment with virtually infinite data storage bulk task computation and network management support. A layered architecture is shown in Figure 1 that demonstrates the interaction of client devices with the centralized cloud. Apart from these characteristics, limitations such as high latency, huge energy consumption, delayed response time, high bandwidth requirements, network load, and operational cost-related factors get increased due to vast traffic route toward the cloud specifically due to the IoT and mobile devices, which is not quite feasible for most of the IoT-based tasks that need a real-time response from the cloud servers [7].

**Figure 2:**
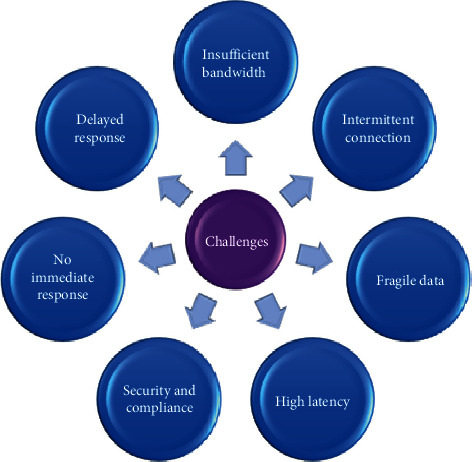
Data processing challenges at cloud data center.

IoT falls under three major categories: consumer IoT, industrial IoT, and civic IoT [8], and most of the IoT applications are delay-sensitive; they need real-time data processing and require results in a very short time. IoT and cloud work together, IoT devices as heterogeneous in nature, generate the tasks in different forms; mainly, these tasks are of three types: data, computation, and application which is contained in a virtualized form known as containers that are light-weight virtualization concept as it is less time and less resource-demanding, so the tasks are transferred from device to device known as task offloading or service delegation [9].  Figure 2 shows the challenges involved in a typical cloud environment that need to be addressed in order to realize the true benefits and ensure a successful user experience. The problem arises when a huge number of data is generated by these devices simultaneously; consequently, the network traffic rush becomes increased, unnecessary bandwidth is consumed, and ultimately other applications especially requiring surrounding information face network latency due to congestion in the network. Normally, these devices are located at dispersed locations as well as at the local level, generate the task, and then receive the desired results after processing at remote cloud; reasonably, no need to offload the data unnecessarily to the cloud; instead tasks must be executed at some local environment. The current traditional centralized cloud architecture exhibits the deficiency of fulfilling the underlying problems. The current approach encompasses moving all required data from the data generated devices to the data center for data storage; computation or network services essentially increase the latency and reduce the response time [10].

Due to the popularity of smart devices, multimedia content, video streaming, and voice services, higher data transmission rates are required. Current communication technologies, i.e., 3G and 4G, are supporting and fulfilling the requirements of ever-increasing IoT and mobile devices but to some extent till the limited device mobility and network coverage. Distributed and heterogeneous data traffic is going to increase gradually and so rapidly that these technologies may not handle them appropriately. The advancement in high-capacity channels, improved latency-related issues, and better data transfer rate has become the critical challenges to obtain the QoS to its desired level. Being an emerging standard, 5G is a promising communication technology that claims to achieve the desired goal of enhanced QoS.

Undoubtedly, 5G performs relatively better than other technologies in reducing too much latency in sending and receiving the information over the WAN assisting in developing and managing applications with ever better devices and services. Problems arise with delay-sensitive applications that need a real-time response from the servers to perform their intended operations as per their resource requirements. These include Unmanned Ariel Vehicle (UAV), remote surgical operations, image processing, and natural language processing systems. Alternatively, it can be said, IoT and 5G may go parallel when mentioned issues would have been resolved. As far as 5G is concerned, imagine, apart from the 100 times faster technology with improved latency, too many tasks are offloaded to the cloud would result in network congestion with increasing bandwidth problem, eventually remaining to create the latency problem.

The ultimate solution to analyze and process most of the data is the placement of a middleware entity where the necessary operations can be performed as per requirements near the IoT devices. Sensing the problem, other middleware entities like cloudlet, fog computing, Multiaccess Edge Computing, Femto-cloud and Delay Tolerant Network (DTN), etc. were introduced given that a service provider offers to depend upon the applications' requirements. The basic purpose of this middleware is to facilitate the IoT devices near the origin of the tasks that require local processing, so a prompt response may be provided accordingly [6, 11, 12]. This middleware resides between the IoT devices and the cloud data center that receives, gathers, analyzes, and evaluates the tasks by applying dynamic offloading policies and decides whether the task needs to be processed at some local levels or at the cloud.

### 1.2. Middleware Technologies

A cloudlet, mobility-enhanced small-scale cloud data center, was introduced by Carnegie Mellon University (CMU) as a research project aimed at providing computational and data storage resources to make the devices nearby instead of accessing over the Internet traditional cloud and proposes overcoming some of the issues like high latency, substantial response time, and data loss that mobile cloud computing architecture faced. In order to make efficient use of IoT resources such that saving battery, reducing processing power, and keeping enough storage, mobile users can offload their tasks to these cloudlets. Cisco extended this concept by arguing instead of having just one entity that provides services on a small scale that is too resource deficient to generate intensive applications, i.e., machine learning-based intelligent applications, high-performance computer applications, smart healthcare, and smart city applications; rather, a resourceful entity is required, quite reliable whenever it needs to offload; that entity is available and can perform extensive tasks [6, 12]. For that reason, Cisco proposed a three-layer architecture in which distant cloud finds its place hieratically at the top level and IoT devices are located at the physical layer where all underlying nodes use the applications and services [13]. A middleware entity would perform the necessary localized tasks generated by the IoT devices while eliminating and reducing the delay factor and personalizing the services as well. If, however, tasks need to offload to distant cloud data centers, this signifies this paradigm which was not possible in cloudlets. This new paradigm “fog computing” overcomes the cloud limitations while providing such a computing environment where a requesting device can be entertained at some local level closer to the IoT devices near the data generated source [14, 15]. Fog, as an emerging computing paradigm, assists the cloud in handling the huge amount of data generated by the IoT devices and processing the data closer to the source where it is generated as well as coping up with the security challenges. On the other hand, this technology is perceived at the initial stage and much work needs to be performed so far. It is quite pertinent to mention that this new paradigm fog computing is not the replacement of the cloud; rather, it fulfills the computational requirements of the devices at the local level where the data is generated and the processed data may be offloaded to the cloud (if requires) and back to requested devices [16, 17].

### 1.3. Fog Computing

**Figure 3:**
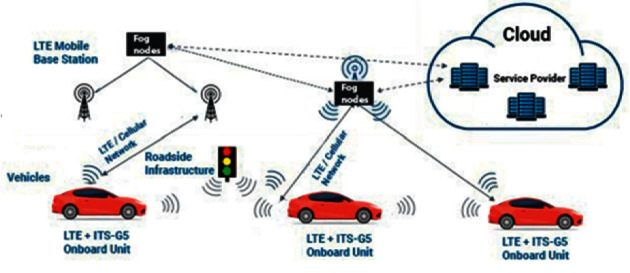
Smart car parking system.

Different IoT and mobile applications need a diverse set of resources to be processed as per their task requirements. Requiring resources vary from application to application; among them, different applications require computational resources; on the other hand, some have delay-sensitive characteristics, so in this way, these applications can be categorized into two classes: one falls under delay-sensitive applications, and second falls under computation-intensive applications [18]. For example, in a smart car parking system, smart cameras are installed in the parking areas as shown in Figure 3 that sends the captured pictures to the fog nodes after every five seconds; an image processing system, implemented at the fog node, acquires each picture, analyzes it as per morphological processing measures, identifies the status of the slots, either vacant or occupied, and updates the actuator accordingly [19]. In a smart waste management system, Ultrasonic sensors are equipped with each bin to know the status of the bin level. The percentage of waste level is measured and data is sent periodically to the concerned application modules installed at distributed fog nodes and the placement and communication mechanism for each waste department is informed by these modules at the fog level [20].

In a smart mining system, because of the unpredictable hazardous in nature and a dangerous environment for human beings even in normal situations, multiple sensors can be associated with the fog nodes to know the status of the environment as land sliding may happen anytime or any harmful unforeseen event that may not be anticipated.

Various application modules in the mining system need to be created at fog nodes to receive and process the data instantaneously as per their processing requirements. Suppose that sensors including gas, chemical, and surrounding sensors are used to identify the status or presence of natural resources; fog modules will take into account the values captured by the sensors and process and generate a response, respectively [21]. Because data is generated, analyzed, and transferred at different levels in all three above-mentioned scenarios, concerning applications, modules play their intended roles with the data and produce the desired results that could not be possible at the device level. Hence, computation-intensive applications can be entertained at the fog level. It is worthwhile that these devices need the response with respect to their current location and the scenarios demonstrate the benefits of fog computing; tasks are not required to send to a central cloud since these tasks can be handled at the fog level effectively and efficiently, within the proximity of IoT nodes and computational resource in fog; hence, the reduction in latency and communication cost is achieved for computation-intensive applications and services [22]. These fog nodes can send the processed data to the cloud for either permanent storage, analysis purpose, or further centralized processing, etc. Task response time becomes improved due to the immediate availability of fog nodes [23].

### 1.4. Advantages of Fog Computing

Fog computing is based on such computing architecture where fog servers (nodes) are providing a diverse set of services like cooperation, the interaction of technological components, and communication services among connected vehicles, patient monitoring systems and traffic signals, etc. ubiquitously in a distributed manner. Instead of contacting the centralized cloud, fog enables the client devices to utilize its resource pool at the edge of the network with the scalability characteristics in ever-increasing IoT and mobile devices [24–26].

### 1.5. Fog-Cloud Collaboration

Although fog computing has proved to be a promising standard paradigm that provides services to various IoT and mobile devices at the network edge, a lot of research challenges need to be addressed as the topic of the day. In view of the fact, fog is heterogeneous in nature by means of node's capabilities while residing inside the IoT domain, reaching the desired performance level, computing resource provisioning in terms of task offloading, and optimal response time with improved latency are some of the exemplary research challenges [27].

**Figure 4:**
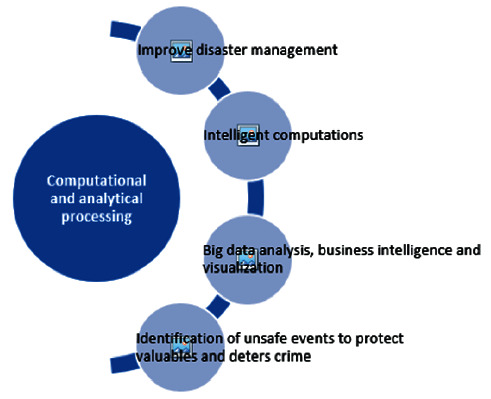
Computational and analytical processing.

It is worthwhile to mention that all these research challenges reflect the Quality of Service (QoS). Besides, it is admitted reality that the current network topology and approaches in a traditional remote cloud computing paradigm for managing the data are no longer feasible to deal with such huge data. Research in these areas is being carried out in a quite recent in which fog architecture is reviewed with the integration of ICT characteristics and support. Nonetheless, the challenges of offloading, resource management, and performance level achievement could not be addressed effectively as all the above-mentioned challenges directly affect the QoS. If needed to achieve so, a formal and optimal approach is required to deploy accordingly. However, improving QoS seems to be a critical challenge, particularly when a massive amount of raw data is being generated by mobile and IoT devices that need to be processed within a given period of time but then again what, where, and how. As it is depicted in Figure 4 that an ideal cloud data center contains virtually infinite resources, this massive amount of raw data may be offloaded to the cloud; then, latency-related problems will arise. In case the task is offloaded to the fog environment, the aforementioned problems would be solved due to the existence of fog nodes in the proximity of data-generating devices [28]. Since fog nodes have a limited number of resources, some data and computation-intensive applications may not be entertained at all due to the limitations of fog resources. Here, cloud and fog intersect each other; cloud overtakes in terms of delay performance. It seems that it is dependent upon certain parameters like task length, the computation required, fog capacity, etc. It depends on the priority of the applications whether these applications are delay-sensitive or computation-intensive. There would be a point that distributes the task offloading between two network entities, i.e., cloud and fog. It offloads some tasks to the fog and the rest of the tasks to the cloud. If it is comfortable, then, it is fine; otherwise, fog needs to be enhanced in terms of capabilities.

It is observed that the data generated by IoT can be processed at three levels: local level (on the edge of the devices where it is generated), fog level, and the cloud level. Some processing may be done at a device level, but the fundamental problem is what could be a suitable computing offloading model to improve the QoS at the fog-cloud paradigm that still needs to resolve. Problem scenarios may exist, the first problem is raised what to offload: data, computation, or application, second, where to offload the task, either at fog or cloud level as some tasks really need to be processed at either fog or cloud server(s) but they offload to a wrong computing platform, and third, how much to offload. Usually, the offloading decision is based on certain parameters like task size, instructions per second, uploading, downloading bandwidth requirements, burst (computation) time, etc. These relevant parameters shall be incorporated in respect of these offloading decisions; on the basis of these parameters, an intelligent task offloading model is proposed. After taking the offloading decision at the fog level, which fog server will be responsible for providing the resources to a particular task also represents a question mark.

### 1.6. Task Offloading

The requirements from the IoT and mobile devices are highly unstable and volatile that may not be expected or anticipated straight away associated with the uncertainty of the fog resources; therefore, the problem needs to be addressed. The basic purpose of this research is to propose such cost-effective offloading and resource scheduling predictive model to optimize the given cost of these devices running their applications to use resources for offloading a task, so here all three above-mentioned problems will be addressed in this research article; we bring the recommendations of how to resolve them. Furthermore, some tasks share the same fog resources; the result encompasses resource conflict in certain scenarios that may lead to deadlock, and some tasks face delayed response or there may be the possibility that new tasks will not acquire resources at all; hence, latency makes its place there. It must be resolved to enhance the fog performance by offloading certain tasks to nearby nodes to make sure of the fair utilization of the underlying resources [29].

**Figure 5:**
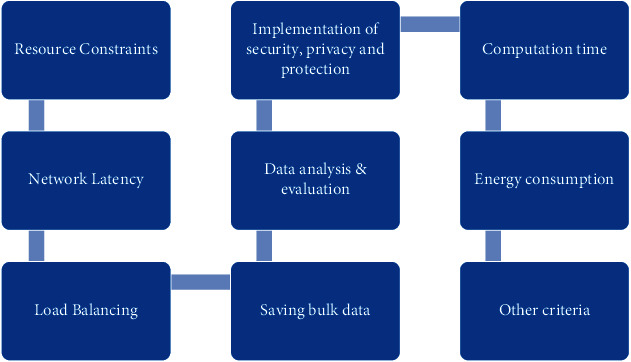
Task offloading criteria.

Some criteria/key reasons for offloading the tasks are depicted in Figure 5. Keeping in view, in spite of these criteria, many other factors like energy constraints, limited bandwidth, not enough computation capability of the servers, insufficient storage space, and task size are also responsible for occurring the offloading process. An offloading management system (OMS) will be responsible for receiving, analyzing, transferring, and monitoring the whole process in this proposed model.

When an application needs to perform certain operations on the tasks being generated, often it requires computation power more than the capability of its source device; certain tasks must be offloaded to a comparatively resource-rich device [30]; for example, a GPS enabled mobile application needs the services of the global map located at the cloud data center.

A task offloading and scheduling problem is presented in [31] where Rahbari et al. addressed the NP hard problem. They proposed a novel Module Placement method by CART (MPCA) algorithm. Task offloading decision takes place on the basis of the following parameters:

- Authentication
- Confidentiality
- Integrity
- Availability
- Capacity
- Speed
- Cost

No doubt, the response time and performance would be improved; however, this approach contains some drawbacks. A decision made to offload the task to either fog or cloud is taken at the fog level, which severely affects the performance of latency-sensitive applications that require the least delay. Moreover, when no fog device executes the module, then the task is offloaded to the cloud because the current status of fog devices has not been observed prior to which fog device(s) is busy. Does a fog node contain adequate resources a task is supposed to use in order to fulfill its computing requirements? Likewise, there is no provision if the task may be offloaded to the cloud in case of using cloud resources afterward.

Park et al. proposed a dynamic task offloading model in [32] using deep reinforcement learning (DRL) based algorithm. The proposed algorithm is intended for joint optimization of delay and energy-efficient communications. The article focuses on both delay and energy consumption at each terminal device using the offloading parameters including processing and transmission delay as well as the processing and transmission energy at both terminal and edge servers for the reduction in execution delay and improved energy consumption while satisfying tolerance delay and a trade-off between execution delay and energy consumption. Keeping in view the fact that the tasks generated by these devices exhibit diverse characteristics like device connection, data sensing, data transport and access, etc. may not need the same computing resources all the time. More training would be required in this ever-changing and digital transformational environment but too much reinforcement learning may result in spoiling the working performance of the model; moreover, the proposed solution needs more data and significant computational power for the agent to learn a close optimal policy, which increases the cost and complexity of the model correspondingly.

Even though the paper improves the task offloading performance by employing DRL using two parameters, i.e., delay and energy consumption, other significant communication-related performance improvement factors such as latency, bandwidth, response time, and cloud communication are not discussed. In case the proposed solution is directly set up at a fog-cloud collaborative system, the desired performance would not be achieved due to the lack of mentioned parameters along with the absence of a cloud data center. The reasons include changed environment settings or reward decision parameters that are required to be updated periodically. Various factors need to be taken into account like network allocation, network status, and resource availability as well as the hybrid nature and scope of heterogeneous devices with the real-world implementation. Since edge computing is a centralized solution for providing better task offloading and data processing, on the contrary, the underlying devices are located at some scattered remote geographical locations; therefore, it is strongly recommended and pragmatic that the computing environment is configured in a distributed manner.

There is no doubt that reinforcement learning is a machine learning framework employed to solve various sequential decision-making problems that enable an agent to evaluate the current environment to maximize returns; too much reinforcement learning may lead to an overload of the states that diminishes the training results. Some limitations are found in terms of the offloading decision at the device and mobile edge computing (MEC) level. When the offloading process occurs, the tasks are offloaded to MEC servers regardless of the current status of the MEC network. For example, edge servers either are busy or do not contain adequate resources and how it is possible for the offloaded tasks to be entertained at which server and/or rest of the tasks may be offloaded to some other entity say cloud data center, etc. that contains enough resources required by that particular task.

Another task partition and scheduling algorithm (TPSA) is proposed by Li et al. in which an Artificial Intelligent (AI) based collaborative computing mechanism is proposed, which is based on deep deterministic policy gradient using deep reinforcement learning technique [33]. Computational cost and failure of service can be reduced along with improving the reliability and allocation of workload and scheduling the order of execution of task offloading in vehicular networks. On the other hand, the proposed algorithm does not provide the solution to assess or compute the optimal computing strategy. Furthermore, it does not cater for computational-intensive applications that, in case, cannot be entertained at some edge servers and may be denied before or during the execution process.

In order to maintain the interactive and streaming nature, delay-sensitive applications like patient monitoring systems, voice over IP, disaster management applications, and multimedia hosting services, where the process is only possible on the basis of live streaming, require a low end-to-end delay. Hence, the set of tasks or part of the task must be offloaded to a certain environment residing nearer to the proximity of the devices where the whole task or the contents of the task can be processed within an acceptable timeframe [34, 35].

### 1.7. Research Contribution

The underlying research article concentrates on the task offloading in a fog-cloud system with the intention to enhance the user experience and QoS together with making the best use of the resource utilization by employing the best policy practices. The proposed work will achieve significant-factor efficiency in respect of computation time, latency, energy consumption, bandwidth, and total network utilization. The intelligent offloading manager is responsible for taking offloading decisions based on the policy it contains; it predicts in terms of anticipated operations in the whole setup. As a key note of this research article, the contributions would be as follows:

- An intelligent task offloading model is proposed in fog-cloud collaboration based on logistic regression, a machine learning algorithm, which predicts the task offloading and the status of resource utilization. Multilevel task offloading, in which the process is performed at (i) IoT and mobile devices layer, means no offloading, (ii) device-to-fog layer offloading, and (iii) device-to-cloud layer offloading.
- Dynamic Offloading Model will solve the problem intelligently what to offload: data, computation, or application, where to offload the tasks: fog or cloud platform, and how much to offload.
- Work performance, anomalies (if any), and the rationale of how significant the operational functionality of the proposed framework exhibits will be evaluated.
- The proposed model is a three-layered architecture where the offloading process is classified into three levels and the rules of the offloading process begin when a device cannot find the desired resources to perform its intended tasks at a specified layer.

As a proof of concept, the proposed model is implemented by employing a simulated environment using a machine learning-based solution to fully demonstrate the success and realization of the proposed solution of the task offloading for fog-cloud resource utilization efficiently maintaining load balancing while reducing the overall latency, energy consumption, response time, bandwidth, network load, and operational cost to the maximum. Simulation results will prove the significance of the robustness of the proposed model demonstrating the potential to maximize fog computing prospects.

The rest of the article is organized as follows. In Section 2, the related work (literature review) is reviewed. The proposed model is presented in Section 3 with a machine learning-based intelligent offloading mechanism and results.  Section 4 presents interpretation of results. Finally, in Section 5, the article is concluded and presents the future research directions.

## 2. Literature Review

Cloud-based computing presents certain service and deployment models that transform software integration, strategic accessibility, and synchronization. While reducing the IT cost, it participates in business continuity, the flexibility of work practices, and access to automatic updates. More specifically, intelligent and accurate resource management provided by significant contemporary technologies like AI attracts the interest of the research community serving in various fields using cloud computing. On the contrary, there is no doubt that IoT and mobile devices enhance the computational capability and service range with the assistance of these contemporary technologies in business automation, smart decision-making, improved customer experience, research and data analytics and medical research, etc. However, some key challenging issues have been raised with the advent of these devices as an enormous amount of data is being generated on a continuous basis that, preferably, need to be processed promptly and these devices are resource-constrained like power hungry having low computational power, less storage capacity, and delay intolerant portable devices which is not enough appropriate to resource utilization [2]. Tasks need to be performed, for example, at some nearby network's edge in a close proximity to these devices. Many researchers have proposed different models for task offloading in their respective field of interest.

For solving the task offloading problem, a four-tier architecture is proposed in [5] with the game theoretical approach on the basis of time (delay) and energy consumption as parameters. Since the analysis of this game is performed in the form of an evolutionary game, the Maynard replicator dynamics has been employed as the dynamic routine for the proposed evolutionary game. As a real dataset, tasks are collected using Python scripts and simulated multiple scenarios in MATLAB. It is a good contribution to the task offloading process; on the contrary, a delay gets increased due to the increasing number of tasks that need to be improved.

Aazam et al. proposed architecture in [11], which consists of three layers with a global gateway: fog only, fog-cloud integration, cloud only (F/FC/C), and GG by evaluating three-tier COT architecture taking into account various applications using real datasets based on three scenarios for offloading: fog-only, cloud-only, and fog-cloud collaborative. As far as cooperation mechanism in fog computing is concerned, all servers in fog cooperate with each other and then decide who will perform certain tasks. Even in the presence of a gateway, power consumption and unnecessary load would be increased.

Liu et al. optimize the max-min fairness of energy balance, which aims to achieve the fairness of the energy among multiple IoT devices by optimizing communication, computation resources, and computing mode selection in [15], and adjust the frequency of CPU according to the nature and scope of IoT devices. There are three layers in the proposed model with the divisible task offloading process. If offloading often takes place, then latency would be reduced as well as the reduction in energy of the devices accordingly. Wireless-powered hierarchical fog-cloud computing network is studied where IoT devices are charged by a Hybrid Access Point (HAP) as well as processing their computation tasks. A Generalized Benders Decomposition (GBD) based solution method is presented at first to find the globally optimal solution. It uses a three-tier system model consisting of IoT devices, Hybrid Access Point (HAP), and cloud center. (HAP contains the Fog devices: servers, etc.). All offloading including fog and cloud will be done via HAP and HAP provides Wireless Power Transfer (WPT) to IoT devices. Research work lacks how to distribute the tasks at different servers in fog, cloud, or both fog-cloud collaboration. Moreover, the criteria of the poor, worst, good, and best channel gain are not mentioned.

Jošilo and Dán addressed the problem in [27] of how devices can improve their performance by offloading their computational tasks at a nearby device or edge cloud. Their contribution involves the analysis of fog computing systems and coordinating collaborative computation offloading with low signaling overhead. They proposed an efficient decentralized algorithm based on an equilibrium task allocation in static mixed strategies. To compute an equilibrium task allocation in static mixed strategies, a game-developing model was employed using variational inequality theory. A decentralized algorithm is proposed in this paper for allocating the computational tasks to the desired locations that will be demonstrated by the simulation process. The algorithm is compared with a myopic algorithm. Two models are presented: Communication and Computation Models. Tasks offloading depends upon certain parameters, e.g, execution time, transmission time, waiting time, response time, latency, size, and complexity of the tasks. A problem in this research work includes the following: standards/metrics or range of these parameters are not mentioned; acceptable time for computation and energy cost of offloading have not been considered in this research work. Output delay is not catered as it was assumed that it could be very small; that is why it might be ignored.

The problem relates to task offloading and resource allocation, based on energy and time-efficient computation in terms of how to optimize the computing/completion time and energy consumption of application requests from IoT devices at the same time, e.g, computation offloading selection and transmission power allocation. Sun et al. in [28] proposed the IoT-fog-cloud architecture that demonstrates the advantages of fog and cloud. In order to improve the energy consumption and completion time of application requests, an algorithm of energy and time-efficient computation offloading and resource allocation (ETCORA) is proposed that is simulated at the paper end to verify the algorithm in reducing energy consumption and completion time of requests. It improves the energy consumption and completion time of application requests. The values of the computing offloading parameters (input data size, task deadline, computing time, transmission power) are not provided; rather, their mathematical equations are mentioned.

With the aim of reducing the vehicular networks service latency as well as improving the reliability, allocation of workload, and scheduling the order of execution of task offloading, a task partition and scheduling algorithm (TPSA) is proposed in [33]. In order to ascertain the policy for vehicle task offloading, an AI-based collaborative computing mechanism, deep reinforcement learning technique, i.e., deep deterministic policy gradient, is devised. By employing this mechanism in collaborative computing, the cost of service and plenty of service failure can be reduced. They claim that the proposed TPSA will be recommended to comply with the low latency and reliable services to the users even in a multifaceted urban transportation network structure. However, the algorithm may not provide the solution to figure out the optimal computing strategy and is left for another research work.

A dynamic task offloading mechanism is proposed in [34] for software-defined networks. In this research, multihop access points are used with fog nodes. In order to present an Interlinear Programming formulation of the problem, Misra et al. employed a linearization technique by proposing a greedy heuristic-based mechanism. Their experiments demonstrated that energy consumption and the average delay are relatively reduced by 21% and 12%, respectively. A problem exists in their work with a static network topology as mobile access points exist in real topologies which they have left for future work. The proposed scheme seems to be a good solution for reducing the average delay as well as energy consumption in the entire network. Changing factors, such as the increasing number of tasks, heterogeneous and dynamically distributed locations of the devices, and accessing gateways/access points to create the additional overall delay, must be incorporated and addressed.

Deep Q Network (DQN) based dynamic offloading algorithm is proposed in a mobile edge computing system in [35]. Since the proposed algorithm is based on deep reinforcement learning (DRL), therefore, it improves its performance on the basis of DQN. It is designed for the joint optimization of both delay and energy-efficient communications. In so far as the performance evaluation of the proposed algorithm is concerned, simulation results prove that this algorithm achieves the desired performance specifically in case the environment exhibits changes/improvements in its computational strength. In order to perform performance evaluation as well as real-world implementation, other parameters like computational overhead, bandwidth, and energy efficiency should be incorporated in mobile edge computing. As far as the proposed algorithm is concerned, it seems good; however, it does not qualify for providing scalable computing characteristics. Design and implementation of distributed computing are left for future work that would contribute to the scalability and practicality of the whole network.

In [36], using the notion of game theory, two QoS-aware distributed algorithms are proposed. For Industrial Internet of Things (IIoT) devices, a Multihop-Communication Cooperative Model (MCCM) and the QoS-aware computation offloading and routing problem are devised as a Multihop Cooperative computation offloading Game (MCCG). In order to restrain the underlying devices acting as relays as well as used potential game theory to prove that the game achieves a Nash Equilibrium (NE), Zicong Hong et al. designed two QoS-aware distributed algorithms that can reach an NE and proved the convergence of the algorithms. Finally, the simulation results demonstrate the working functionality of the proposed algorithm. The proposed algorithm not only balances properly as the IoT device size increases but is more stable and performs better than existing algorithms under a variety of parameter settings.

In order to minimize energy consumption and service latency, keeping an eye on critical challenges of wide deployment of Vehicular Fog Computing (VFC), Yadav et al. proposed an energy-efficient dynamic computation offloading and resources allocation scheme (ECOS) in [37]. While satisfying the vehicular node mobility and end-to-end latency deadline constraints, the ECOS problem is addressed as a joint energy and latency cost minimization problem. On the basis of resource utilization, a computational offloading selection policy is devised that offloads the tasks from an overload cloudlet node. A heuristic approach is proposed between the vehicular node and selected IoT-related tasks that resolve the resource allocation problem. It is claimed that the proposed scheme can effectively minimize the energy consumption and service latency; however, the process of obtaining overall computational capability, resource availability, and communication cost of a particular cloudlet node have not been examined. Moreover, if incoming tasks demand more computation beyond the capability of cloudlet, then the task cannot be processed at all. It is important in view of the fact that the current status of cloudlet nodes needs to be distributed across the network as it becomes more specific in a heterogeneous environment. The mechanism of intercommunication among vehicular nodes is also missing and left for future work.

## 3. Proposed Research Methodology

In this section, the proposed model and the interaction among its components with the essential interfacing requirements are demonstrated. The proposed model consists of three layers in respect of intelligent task offloading in fog-cloud systems. It is composed of both fog and cloud servers. This underlying fog-cloud environment is comprised of distributed resources that are heterogeneous in terms of network hierarchy start from the very basic physical layer of a network to the centralized cloud environment. Heterogeneous means these devices are dispersed at different geolocation and not stationery. The host servers, which perform as computing resources, intended for providing services to various application tasks, are enriched with a diverse set of resources. It is based on two types of applications, i.e., delay-sensitive applications and computation-intensive applications.

### 3.1. Fog-Cloud Intelligent Task Offloading Model

**Figure 6:**
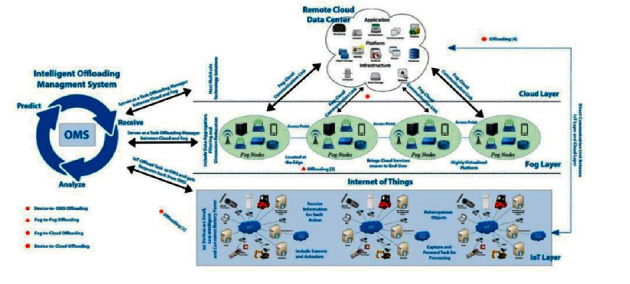
Proposed fog-cloud intelligent task offloading model.

The architecture consists of three layers and an intelligent task offloading management system (OMS) as shown in Figure 6. The following are the brief details of each layer:

- IoT/physical layer
- Fog layer
- Cloud layer

Each layer is composed of different devices, nodes, and servers, respectively. All IoT and mobile (sensors, actuators, and other data generation) devices are located at a physical layer. The fog layer contains a set of servers, normally called fog nodes. The cloud layer contains the set of servers (virtual machines). The proposed model can be understood as a hierarchal model that contains three offloading modes. No offloading mode is performed at the physical layer provided; the devices contain sufficient storage and computation power and run the application on their own. The offloading process begins when a device cannot find the desired resources to perform its intended tasks; initially, an intelligent offloading management system (OMS) is an entry point in an offloading process.

The following are the brief details of each layer.

#### 3.1.1. IoT/Physical Layer

The physical layer is a low-level layer in the hierarchy of the proposed model that consists of all interconnected heterogeneous IoT and mobile devices that generate the data. Data will then be offloaded to the upper layers of the model. For simplicity's purpose, all devices are named that generate the data as sensors, and the devices that provide the results are named as actuators. In this way, the same device may be a sensor or actuator depending on its working behavior. In a smart city, various types of trackers, cameras, LCDs, buzzer alarms, virtual glasses, etc. act as sensors and actuators that enable things to see and perceive their environment, make collaborative decisions, and make corresponding tasks based on the observed data.

#### 3.1.2. Fog Layer

The fog layer is located in the middle of the hierarchy in the proposed model as shown in the middle layer of the model. It is a distributed network environment closely related to both the physical layer (bottom) and cloud layer (top). This layer contains multiple servers called nodes that are distributed across the network. These fog nodes contain the adequate set of resources that an IoT device may demand including data storage, computation, application execution, and placement services. Since it is required to enhance the overall efficiency and reduce the data being routed toward the cloud, therefore, several fog nodes are deployed in this proposed model setup. In so far as the fog layer is concerned, it is meant to improve the overall efficiency of the network, so every task that needs to be processed is expected to be handled at this level. This improves the QoS by lowering down the latency and network response time. Tasks are assigned such node(s) that contain adequate computing resources by fair utilization of these resources.

#### 3.1.3. Cloud Layer

The cloud layer is located at the top of the hierarchy in the proposed model; that is, unlike fog computing, the cloud data center is centralized in nature. As is mentioned above, a typical cloud server has virtually infinite computing resources; all resources are assigned that an IoT task can demand. The servers located at the cloud layer can run extensive applications like machine learning-based contemporary applications, natural language processing, big data analytics, etc.

### 3.2. Offloading Management System

An entry point in an offloading process that possesses four main characteristics:

- Custodian of Offloading Policy Repository
- Hold the recent status of fog snapshot
- Receive-analyze-offload the tasks
- Prediction construct

Brief details of the characteristics of the offloading management system are described hereinafter.

#### 3.2.1. Custodian of the Repository: Task Offloading Policy

Conventionally, an OMS contains the policy details about the whole offloading criteria. Which task needs to be offloaded to which network and based on some criteria as well as what strategy would be implemented in which situation and how as it is a complex process to perform with the underlying decisions?

#### 3.2.2. Hold the Recent Status of Fog Snapshot

An OMS is linked with the master node of a fog layer in an asymmetric environment which knows the status of all fog nodes and the availability of the free resources. The master server sends the OMS about the details after a specified interval of time. The master server distributes the tasks among fog nodes according to the resources they contain and return them back to the OMS after receiving from the fog nodes.

#### 3.2.3. Receive-Analyze-Offload the Tasks

**Figure 7:**
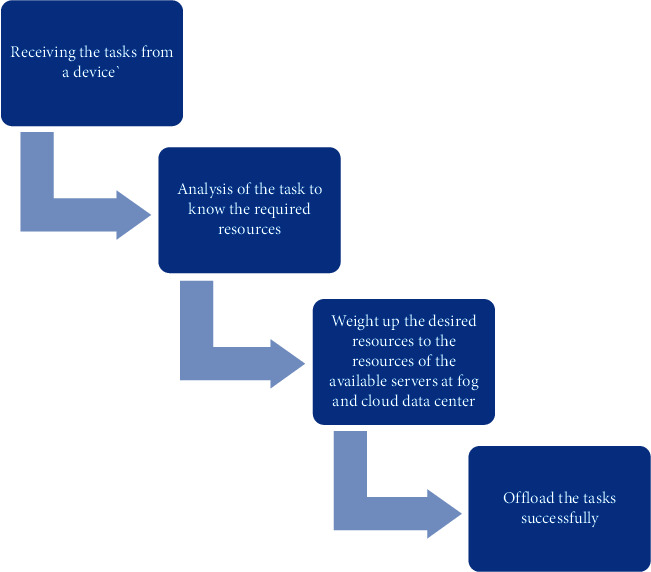
Task offloading procedure.

IoT and mobile devices are supposed to send the tasks to the network for processing purposes. That task may consist of data that needs to be sorted, stored, video streamed, or rendered. It may need computation to be processed accordingly. A task may be an application that needs to be offloaded at some nodes. After receiving the tasks from a device, OMS is responsible for offloading the tasks to the concerned computing platform, so according to the policy it follows, the whole procedure of task offloading is explained briefly in Figure 7.

#### 3.2.4. Prediction Construct

Usually, a large number of recurring tasks are generated, which are similar in their resource requirements, frequently needing to be decided from scratch for offloading process would be causing unnecessary delay in offloading decision-making. The traditional offloading mechanism is not quite suitable for the success of the entire process in terms of assurance of QoS. Machine learning is a form of an automated data analysis for developing analytical models [38]. That is why it enables the task offloading process to access hidden patterns, trends, and insights of the received tasks. It improves its working functionality continuously from the past data. Being a predictive analysis machine learning algorithm, logistic regression is used in this article to classify the tasks based on probability. Application scenarios are continuously changing with the usage and the scope of their tasks becoming updated gradually; it is quite feasible for an OMS to be trained as it improves the learning capability of the overall network. Although it has been observed in presenting offloading decision process by employing some traditional machine learning algorithms, deficiencies in the current machine and deep learning methods include slow learning speed, may not cope up with the ever-changing environment, and input data from diverse sources, together with simplifying and integrating offloading scenarios and testing protocol. Due to ever-changing scenarios and requirements, logistic regression is used in the proposed model in the IoT-fog-cloud environment to perform the actions relevant to predicting the offloading decision. The objective is set for this so-called offloading manager to choose actions that maximize the throughput within a given amount of time.

### 3.3. Categories of Task Offloading

**Figure 8:**
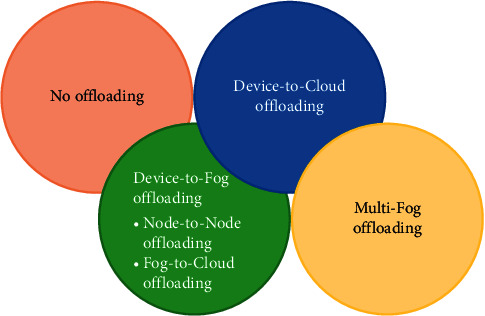
Task offloading categories.

The entire process is activated by the intelligent OMS. Its formation consists of offloading policy repository, organization, and monitoring offloading process, a recent snapshot of fog capability and availability, and knowledge base. It organizes each task and analyzes and offloads according to the optimization policy.  Figure 8 depicts the four formal categories of task offloading. The following section explains the proposed categories of offloading process.

#### 3.3.1. No Offloading

A task offloading process is not required when the task is no longer supposed to offload and obtains the desired resources from the task generating device. For example, a simple comparison between two values does not need to be processed at some remote location; rather, it would be performed at the device by utilizing the resources of the same device. Normally, no offloading is quite appropriate for nonsharing datasets that can be handled at the device level.

#### 3.3.2. Device-to-Fog Offloading

The offloading process starts when the task needs to send to some remote location or a local device cannot find the required resources to process the task data itself. The task is offloaded to either fog or cloud in the setup and it would be done using an OMS installed at the entrance of the fog environment.

*(1) Node-to-Node Offloading*. Once the fog network is selected for offloading the task, it becomes significant to which node that specific task needs to offload since several fog nodes are providing their services in a typical fog layer exhibiting different diverse resource provisioning capabilities. It falls under load-balancing characteristics too in terms of offloading the tasks to such nodes that have not been considered for assigning the tasks while other nodes are busy with the processing of already assigned tasks. It is significant to assign the tasks to such nodes that conform to the resource availability for that specific task(s). Hence, certain criteria may be taken into account as the current location of the node, service status, rating of processing the tasks, required resources-richness, low latency, improved response time, etc. Furthermore, if the task is dividable, then the task may be offloaded to various nodes.

OMS contains the updated status of all fog nodes in respect of mentioned parameters. Whenever the current system state is changed periodically, the concerned node is intimate to the OMS about its transitional situation accordingly.

*(2) Fog-to-Cloud Offloading*. In some cases, when tasks are processed at the fog level, it is required to offload the resultant data to the cloud level for performing further operations on that processed data or storing permanently. The cloud analyzes the data after receiving it from fog nodes and processes it accordingly by either sending back the responses to fog nodes or storing them correspondingly.

#### 3.3.3. Device-to-Cloud Offloading

Tasks may be offloaded directly to the cloud. The rationale involves here encompassing and storing the data permanently without requiring the fog resources (reasons may be not enough storing and/or computational capacity). Security and privacy issues are considered to be important factors for IoT device-to-cloud offloading process.

#### 3.3.4. Multifog Offloading

Multifog offloading occurs when a task needs to offload to a specific fog network and it is busy or may not comply; then, the fog-to-fog offloading process is activated providing a nearby fog network which is available and permissible. The tasks of autonomous vehicles, drones, and mobile devices usually need such offloading to perform their intended operations.

### 3.4. Simulation Setup

Apparently, it seems that the method that is performed in [11] using SFogSim simulator is a traditional way to tackle the task offloading problem. On the contrary, as far as the delay-sensitive specifically AR/VR and real-time applications are concerned that need a prompt response within a prescribed range of time, such mechanism is required that fulfills the true requirements of these applications efficiently. Requirements include low latency, improved response time, enough bandwidth, and less energy consumption. The machine learning simulation environment is performed to assess and appraise the proposed fog-cloud intelligent task offloading model. The simulation has been performed using Anaconda Python which is a data science platform for data scientists, IT professionals, and business leaders. It is a distribution of Python, R, etc. The model is trained using a set of machine learning algorithms which include logistic regression algorithm, K-Nearest Neighbor (KNN), Naïve Bayes, Decision Tree, Support Vector Machine (SVM), Multilayer Perceptron (MLP) while logistic regression algorithm is the proposed algorithm.

The dataset is obtained from [11] comprising the tasks that are heterogeneous in nature containing high variability in terms of data types, configuration, and format. Since it is not relatively easier with traditional methods to predict or anticipate this heterogeneous data to meet the intended requirement of delay-sensitive and real-time applications, the dataset contains the necessary parameters that participate in the task offloading decision intelligently, hence maintaining the QoS efficiently while enhancing the user experience. Tuples are classified as abrupt, bulk, large, location-based, medical, multimedia, and small textual data type that contain various time features like Tuple Elapsed Time, Tuple Initiates Time, Tuple Propagation Time, Internal Processing Time, and Total Time Taken by a tuple. Devices, which are located at some distributed places and as mentioned above generate heterogeneous data, are of various types including actuators, dumb objects, mobile, node, and sensor through which these tuples are being generated. In case the task can be entertained at some fog level, it will offload to the fog node providing the given policy accordingly; the target label IsServedByFC would possess the value as 1; otherwise, 0 if the task will be offloaded to cloud data center.

Once the prediction process is finalized in respect of the fog network, the offloading management system (OMS) offloads the task to the designated fog node. On the other hand, according to the mentioned policy, a task would be offloaded to the cloud data center if the underlying task cannot be offloaded to the fog network due to the limitation of the computing resources desired by that particular task. The description of the underlying attributes and their types is mentioned in Table 1.

#### 3.4.1. Cloud Data Centers

A number of servers exhibiting different hardware and software specifications are available at the data centers that are used in the setup as shown in Table 2 which create virtual machines and provisioning of computing resources as services accessed and availed by those tasks that might not be handled at some fog level.

#### 3.4.2. Fog Nodes

A number of 5 fog nodes are used in the simulation process.  Table 3 contains the hardware specification of these nodes. As mentioned above, cloud servers present virtually infinite computing resources as virtual machines process the tasks on the resources that are virtual in nature too, which have been reserved generally, so the tasks that could not be processed at fog level will be processed at cloud data center accordingly. Upon completion of the process, the response would be sent back to the OMS for further operations.

#### 3.4.3. Simulation Steps

Dataset requires some preprocessing operations where details are mentioned below. Data is divided into two main categories, one is intended for training purposes, and the rest of the data is used in the testing and validation process. Approximately 70% of data is used for training purposes. The proposed model is logistic regression by which the model will be trained and predicted accordingly. The symbols used in the simulation are defined in Table 4.

The following are the necessary steps that are performed during the whole simulation process. These steps are in formal nature and must be performed in a predefined sequence of steps.

**Figure 9:**
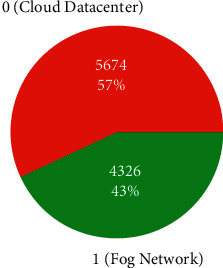
Data bifurcation of fog-cloud offloading.

*(1) Knowledge Discovery in Data*. As far as the discovering and finding of data is concerned, it includes data selection and retrieval, data analysis, and pattern recognition. Table 1 contains 34 features (input variables) and one label (target variable). The target variable contains binary values as 1 and 0. One of the binary values 1 represents that task would be offloaded to fog network, otherwise to cloud data center.  Figure 9 depicts the segregation of rows that correspond to 1 consisting of 4326 (43%) instances; 5674 (57%) instances are of 0 values in respect of output (label) variable.

The algorithm shows the pseudocode of the task offloading process. It explains the entire process of finding the probability of whether the task is offloaded to either fog or cloud. It checks and verifies the values of certain parameters and constructs the prediction process. (Algorithm 1).

## 4. Result Interpretation

**Figure 10:**
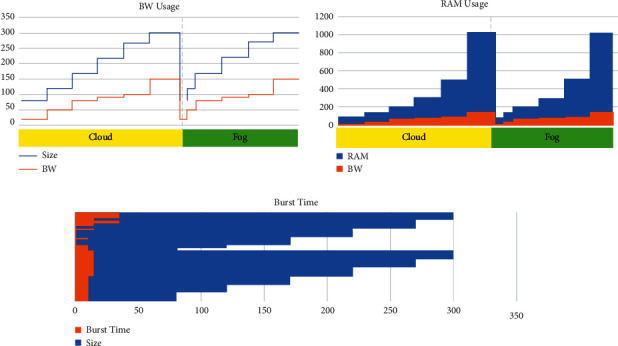
Feature analysis.

Upon observing the dataset carefully, it comes to know that approximately 25% of tasks are offloaded to fog whose size ranges from 80 to 120, 50% of tasks are offloaded to fog ranging from 170 to 300, and the rest of the tasks are offloaded to cloud data center. Tasks are going to be offloaded to both fog and cloud depending on the task requirements and availability of resources for that task specifically.  Figure 10 depicts that bandwidth is going to increase with the task size when it is offloaded to the cloud; on the contrary, it is the opposite in case a task is offloaded to fog and bandwidth is getting reduced. Less bandwidth means reduced delay that improves the response time; hence, network usage and congestion become lower down. On the other hand, memory usage and burst time may not be reduced or affect either task that is offloaded to fog or cloud. A large task needs relatively more CPU burst time compared to a moderate task.

### 4.1. Data Preprocessing

Data preprocessing include feature engineering and feature selection and dimensionality reduction. Feature engineering implicates data transformation, imputation or handing missing/null values, removing outliers, preventing overfitting, dummy variables, standard scaling, etc.

Feature selection and dimensionality reduction is the process of automatically retaining or reduction of the dimensionality of the set of features into the moderate level so that these features can be modeled.

#### 4.1.1. Feature Engineering

Observing the dataset used in the simulation process, it contains missing/null values that need to be handled effectively. As it is not advisable to remove or set mean/median values in place of missing values, therefore, a relevant algorithm is suggested to be used to observe and handle these values so that a formal solution may be devised. Additionally, the services of some domain experts can be taken to resolve the problem correspondingly.

**Figure 11:**
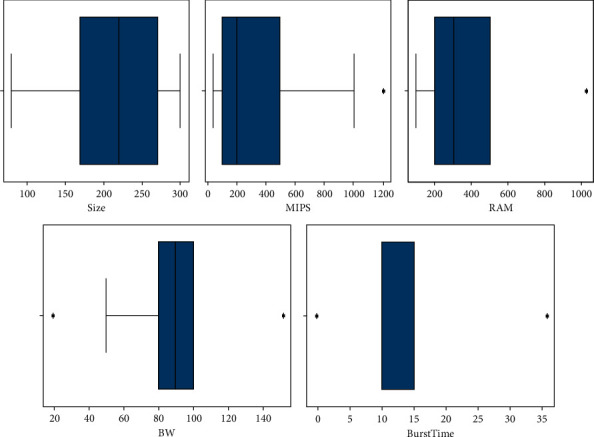
Identification of outliers.

Checking and removing outliers, often datasets contain some values that may be outside the range of the data and affect the expected results. These are referred to as outliers and, sometimes, machine learning exhibits improvements by considering and yet eliminating these outliers. Carefully observing the underlying dataset as shown in Figure 11, some instances in MIPS and bandwidth do not follow a similar pattern to the rest of the data; removing these values is in fact the process of removing the outliers from the dataset. To deal with the outliers in the fog-cloud dataset, techniques include transforming values, deleting observations, imputation, treating outliers separately, deleting/removing those instances that skew the analysis, etc.

Encoding categorical data, machine learning models are supposed to work with numeric values; if the dataset contains some textual data relevant to some categories, then it is mandatory to encode these categorical data to numbers in order to avoid problems. The employed dataset contains the following three attributes with their respective values that are categorical in nature and need to encode accordingly.

- Priority (high, low, medium)
- Data Type (abrupt, location based, medical, multimedia, small textual)
- Device Type (actuator, dumb objects, mobile, sensor, node)

#### 4.1.2. Feature Selection and Dimensionality Reduction

Feature selection is the process of carefully choosing the input variables while planning and developing a machine learning-based predictive model. An approach, the correlation coefficient method, is being described to employ the moderate number of necessary features deprived of compromising the model accuracy by using the correlation method. Feature selection is supposed to be a significant process in the underlying research work that assists in the enormous amount of data being generated by IoT and mobile devices as well as evaluation and management of algorithms in predicting the target network computing system at either Fog and or cloud data center level.

Statistical measures are quite significant to obtain the details of the data that is used to train the machine learning model and to interpret the results in the simulation setup. It incorporates the total number of instances, mean, standard deviation, minimum, maximum, and percentage values.

The Pearson correlation coefficient method is used to select the appropriate features in the simulation process. This method measures the strength of a linear association of variables, specifically two variables by taking a range of values from −1 to +1, 0 indicates no association at all, while −1 and +1 indicate the association of either negative or positive. Hence, the selected features are dependent upon the associated values generated by the Pearson method. The whole working of feature selection and reduction is described in the mathematical formula as follows:

(1) $$\begin{array}{c} \text{r}=\frac{\text{N }\sum \text{xy}-\left(\sum \text{x}\right)\left(\sum \text{y}\right)}{\left[\sqrt{\text{N}}\sum {\text{x}}^{2}-{\left(\sum \text{x}\right)}^{2}\right]\left[\sqrt{\text{N}}\sum {\text{y}}^{2}-{\left(\sum \text{y}\right)}^{2}\right]}, \end{array}$$

where *r* is the correlation coefficient, *N* is the number of pairs of scores, ∑xy is the sum of products of paired scores, ∑x  is the sum of *x* scores, ∑y is the sum of *y* scores, ∑x2 is the sum of squared *x* scores, and ∑y2 is the sum of squared *y* score. Since correlation coefficient is a statistical measure of the degree, statistical measures (count, mean, standard deviation, smallest and largest values of each variable) of the whole dataset are mentioned in Table 5.

### 4.2. Training, Testing, and Splitting the Data

The output of the data preprocessing process is used to split the dataset into two major categories, i.e., train and test. First, the model is trained using the training dataset that is 70% of the whole dataset. Once the model is trained, 30% of the dataset is used for testing the model in order to evaluate the performance of the logistic regression model. Training and testing data are two different but significant parts in machine learning where training data is used to teach the logistic regression algorithm, whereas testing data, as its name implies, assists in validating the progress and optimizing the training of the employed algorithm to obtain the improved results.

### 4.3. Classification Modeling

Logistic regression is used as the proposed model in the simulation process. Some other algorithms are used to train the model. The proposed model calculates the probability of data points of the fog-cloud dataset belonging to either fog or cloud class-related task offloading. The use of exponent in the sigmoid function is validated as the probability greater than zero and the property of exponents takes care of this aspect.

(2) $$\begin{array}{c} \text{ln}\left(\frac{\text{p}}{1-\text{p}}\right)=\beta_{0}+\beta_{1}\text{X,} \end{array}$$

where *p*/1 − *p* is the odds ratio, *β*_0_ is the intercept or bias term, *β*_1_ is the coefficient of *X,* and *X* represents the feature (input value). As far as the training and testing of the data are concerned, the exploratory variables are grouped quantitatively and categorically in the model. Quantitative variables include size, MIPS, RAM, BW, and BurstTime. Categorical variables include Priority, DataType, and DeviceType which are typically referred to as dummy variables, and we can implement a dummy variable multiple regression to obtain these parameter estimates. Here, though our focus is on the dependent variable *Y*, for example, we may be interested in learning if a task offloading would be done at either fog or cloud level; therefore, *y* becomes binary variable taking on categorical values of 1 if task offloading is done at fog and 0 if task offloading is done at cloud.

(3) $$\begin{array}{c} \text{logit }=\text{ log odds,}\\ \text{odds}=\frac{\text{p}\left(\text{fog}\right)}{1-\text{p}\left(\text{fog}\right)}\text{ :}1-\text{p}\left(\text{fog}\right)=\text{ cloud,}\\ \text{X}\in \text{R,}\\ \text{p}\left(\text{X}\right)\in \left[0,1\right],\\ \text{p}\left(\text{y}=1|\text{X}\right)=\text{ p}\left(\text{X}\right),\\ \text{Sigmoid Function p}\left(\text{X}\right)=\frac{1}{1+{\text{e}}^{-\beta_{0}+\beta x}}. \end{array}$$

#### 4.3.1. Task Offloading Prediction

In order to forecast, we need to identify the condition that must be satisfied by the probability function, *f* (^∗^), where

1. *f* (^∗^) must always be positive (since *p* ≥ 0)
2. *f* (^∗^) must be less than 1 (since *p* ≤ 1)

It is required to constrain *p* such that 0 ≤ *p* ≤ 1. Modeling with Linear Probability Model (LPM) where problem includes “can produce predicted probabilities > 1 or  <  0”.

(4) $$\begin{array}{c} \text{Y}=\beta_{0}+\beta_{1}{\text{X}}_{1}+\beta_{2}{\text{X}}_{2}+\;\ldots \beta_{\text{n}}{\text{X}}_{\text{n}}, \end{array}$$

provided that *y* = 1 if at fog level and 0 at cloud level.

*f* (^∗^) must always be positive (since *p* ≥ 0)

Logistic probability function with one explanatory variable can be represented using the exp notation.

(5) $$\begin{array}{c} \text{p}=\text{EXP}\left(\beta_{0}+\beta_{1}\text{X}={\text{e}}^{\beta_{0}+\beta_{1}\text{X}}\right). \end{array}$$

Although this function is always positive, it could be > 1; that is why it will not work.

(^∗^) must be less than 1 (since *p* ≤ 1).

Logistic Regression Function is

(6) $$\begin{array}{c} \text{p }=\frac{\text{EXP }\left(\beta_{0}+\beta_{1}\text{X}\right)}{1-\text{EXP}\left(\beta_{0}+\beta_{1}\text{X}\right)}=\frac{{\text{e}}^{\beta_{0}+\beta_{1}\text{X}}}{1-\left({\text{e}}^{\beta_{0}+\beta_{1}\text{X}}\right)}. \end{array}$$

Not only quotient will remain positive; any number divided by another that is slightly greater than it would always result in a value <1.

(7) $$\begin{array}{c} \text{if }\left\{\begin{array}{c} \text{EXP}\left(\beta_{0}+\beta_{1}\text{X}\right)\text{ is very large},\;p\;\equiv \;1\\ \text{EXP}\left(\beta_{0}+\beta_{1}\text{X}\right)\text{ is very large},\;p\equiv 0 \end{array}\right\}. \end{array}$$

Since the exponent of any number negative or positive is always positive, any number divided by another number that is slightly greater, it would always result in a value of less than one.

(8) $$\begin{array}{c} \text{p}=\frac{{\text{e}}^{\beta_{0}+\beta_{1}\text{X}}}{1-\left({\text{e}}^{\beta_{0}+\beta_{1}\text{X}}\right)}. \end{array}$$

This logistic function is nonlinear; by performing some algebra, the logistic expression can be rewritten to obtain the following linear function, which is the logistic regression model.

(9) $$\begin{array}{c} \text{Therefore},\text{ ln }\left(\frac{\text{p}}{1-\text{p}}\right)=\beta_{0}+\beta_{1}\text{X.} \end{array}$$

Thus, even though the probably *p* is not a nonlinear function of *X*, this transformation is a linear function of X.

In this derivation, the left-hand side of the equation is the natural log of the odds ratios where *p* in the numerator is the probability of success and 1 minus *p* in the denominator here is the probability of failure. The above model is what is implemented in logistic regression.

In the logit model,

(10) $$\begin{array}{c} \text{probability of fog level}=\text{p}\left(\text{y }=\;1\right)=\text{ p}=\;\frac{{\text{e}}^{\beta_{0}+\beta_{1}\text{X}}}{1-\left({\text{e}}^{\beta_{0}+\beta_{1}\text{X}}\right)},\\ \text{probability of cloud level }=\text{ p}\left(\text{y}=0\right)=1-\text{p}=1-\frac{{\text{e}}^{\beta_{0}+\beta_{1}\text{X}}}{1-\left({\text{e}}^{\beta_{0}+\beta_{1}\text{X}}\right)},\\ \text{odds ratio }=\frac{\text{p}}{1-\text{p}}=\frac{{\text{e}}^{\beta_{0}+\beta_{1}\text{X}}}{1-\left({\text{e}}^{\beta_{0}+\beta_{1}\text{X}}\right)/1-\left({\text{e}}^{\beta_{0}+\beta_{1}\text{X}}/1-\left({\text{e}}^{\beta_{0}+\beta_{1}\text{X}}\right)\right)}. \end{array}$$

Odds ratio (relative risk) is the ratio of the probability of offloading the task to fog over offloading to the cloud.

(11) $$\begin{array}{c} \lambda =\beta_{0}+\beta_{1}\text{X,}\\ \frac{\text{p}}{1-\text{p}}=\frac{{\text{e}}^{\lambda }}{{\text{e}}^{\lambda }+1/1-\left({\text{e}}^{\lambda }/{\text{e}}^{\lambda }+1\right)}=\frac{{\text{e}}^{\lambda }}{{\text{e}}^{\lambda }+1/1-\left({\text{e}}^{\lambda }/{\text{e}}^{\lambda }+1\right)}=\frac{{\text{e}}^{\lambda }}{{\text{e}}^{\lambda }+1/1-\left({\text{e}}^{\lambda }+1-{\text{e}}^{\lambda }/{\text{e}}^{\lambda }+1\right)}=\frac{\begin{array}{c} {\text{e}}^{\lambda } \end{array}}{\begin{array}{c} {\text{e}}^{\lambda }+1 \end{array}}=\frac{\begin{array}{c} {\text{e}}^{\lambda }+1 \end{array}}{\begin{array}{c} {\text{e}}^{\lambda }+1-\;{\text{e}}^{\lambda } \end{array}}={\text{e}}^{\lambda }. \end{array}$$

Replacing *λ*, therefore,

(12) $$\begin{array}{c} \frac{\text{p}}{1-\text{p}}={\text{ e}}^{\beta_{0}+\beta_{1}\text{X}}. \end{array}$$

By taking the natural log of both sides, a logistic regression model can be found.

(13) $$\begin{array}{c} \text{ln}\left(\frac{\text{p}}{1-\text{p}}\right)=\beta_{0}+\beta_{1}\text{X.} \end{array}$$

#### 4.3.2. Estimation of the Logit Model

In order to estimate the parameters of the logistic regression model, the Maximum Likelihood Estimation (MLE) method is used.

(14) $$\begin{array}{c} \text{ln}\left(\frac{\text{p}}{1-\text{p}}\right)=\beta_{0}+\beta_{1}\text{X.} \end{array}$$

Estimated probability is p′=*p*/1 − *p*.

After running the logit regression model, the estimated probability is then be calculated, i.e., the probability of success.

(15) $$\begin{array}{c} \text{p'}=\frac{{\text{e}}^{\beta_{0}+\beta_{1}\text{X}}}{1-\left({\text{e}}^{\beta_{0}+\beta_{1}\text{X}}\right)}. \end{array}$$

where *P*′ is the estimated probability of success, and *β*_0_ and *β*_1_ are estimated from the regression.

#### 4.3.3. Interpreting Logistic Regression Result

Two of the four cases are correct predictions, and two are wrong. If *p*′ > 0.5, it would be the case where *y* = 1; else *y* = 0. However, it is safer to use a high cutoff like ≥0.7 to verify the effectiveness of the prediction model.

#### 4.3.4. Interpreting Model Coefficient

(16) $$\begin{array}{c} \beta_{1}\left(\text{y }=\beta_{0}+\beta_{1}\text{X }+\varepsilon \right). \end{array}$$

The sign of the coefficient is interpreted, not the magnitude. If *β* is greater than 0 (positive), then an increase in *X* increases the likelihood that *y* = 1. In other words, it increases the probability of success; i.e., an increase in *X* makes the outcome of 1 more likely. Conversely, if *β* < 0 (negative), then an increase in *X* decreases the likelihood that *y* = 1. In other words, it decreases the probability of success; i.e., an increase in *X* makes an outcome of 1 less likely. The magnitude cannot be interpreted using the coefficient because the different models have different scales of coefficient. Nevertheless, logit and probit models produce estimated probabilities that are fair close.

The estimated likelihood is observed whether the task is offloaded to fog or cloud giving the values in the *X* variables. In the following, the linear model is specified.

(17) $$\begin{array}{c} \text{Y}=\beta_{0}+\beta_{1}{\text{X}}_{1}+\beta_{2}{\text{X}}_{2}+\;\ldots \beta_{\text{n}}{\text{X}}_{\text{n}}+\varepsilon \text{.} \end{array}$$

If *Y* = 1, the task is offloaded to fog, otherwise, to cloud.

(18) $$\begin{array}{c} \text{Y}=\beta_{0}+\beta_{1}\text{Size}+\beta_{2}\text{MIPS}+\beta_{3}\text{RAM}+\beta_{4}\text{BW}+\beta_{5}\text{BurstTime}+\varepsilon \text{.} \end{array}$$

After simulation, the estimated logit model (log of odds ratio) is

(19) $$\begin{array}{c} \ln\left(\frac{\text{p}}{1-\text{p}}\right)=\text{Y }=\beta_{0}+\beta_{1}\text{Size}+\beta_{2}\text{MIPS}+\beta_{3}\text{RAM}+\beta_{4}\text{BW}+\beta_{5}\text{BurstTime.} \end{array}$$

It is worthwhile to mention that the estimated logit model is the log of odds ratio to find the estimated probability of offloading destination, i.e., fog or cloud.

#### 4.3.5. Estimated Logit Model

It is pertinent to mention that ln(*p*/1 − *p*) is the log of the odds ratio, not the probability (*p*); therefore, only the SIGN is interpreted and not the magnitude of the coefficient in a logit model.

### 4.4. Result Interpretation

The results enumerate a substantial advantage in employing a logistic regression model when the response (label) variable is categorical in nature. The advantage has been measured in terms of high accuracy with a low error rate.

Training confusion matrix contains 7000 instances, whereas testing confusion matrix exhibits 3000 instances that fall under different classes. The performance of the logistic regression has been evaluated using the performance measures. These measures present the informative notion of the effectiveness of how much the employed algorithm is efficient. When a task is offloaded to fog classified correctly, it falls under true positive (TP) class; training and testing instances include 3996 and 1678 instances, respectively; likewise, when a task that needs not to be offloaded to fog and classified correctly falls under the true negative (TN), training and testing include 1997 and 866 instances. On the contrary, when a task offloading is misclassified to fog layer, it is false positive (FP); 456 and 1007 instances are related during training and testing processes, respectively, and are referred to as Type-I error, and when a task is offloaded to the cloud misclassified, it is a false negative (FN) with 0 value and referred to as Type-II error.

**Figure 12:**
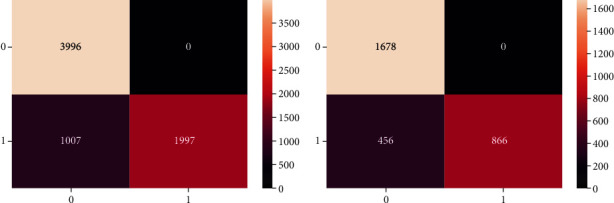
Training and testing confusion matrix.

The confusion matrix consists of training and testing processes as shown in Figure 12 for visually demonstrating the significant predictive analytics where the performance of logistic regression is summarized. It provides direct comparisons among the values of true positives, true negatives, false positives, and false negatives. In view of the fact that only classification accuracy is not enough, several measures need to be taken for evaluating the performance of the algorithm that are as follows with their corresponding formulas. Accuracy can be obtained as the ratio of training instances correctly identified to the total instances in the fog-cloud dataset.

(20) $$\begin{array}{c} \text{accuracy}=\frac{\text{TP }+\text{ TN}}{\text{TP }+\text{ TN }+\text{ FP }+\text{ FN}}. \end{array}$$

Precision shows the ratio of detection that is correctly identified to the total instances.

(21) $$\begin{array}{c} \text{precision}=\frac{\text{TP}}{\text{TP}+\text{FP}}. \end{array}$$

Recall (sensitivity or true positive rate) is the correct detection ratio to the total number of actual detection cases in the dataset.

(22) $$\begin{array}{c} \text{recall }=\frac{\text{TP}}{\text{TP}+\text{FN}}. \end{array}$$

*F*-Score characterizes the trade-off between the precision and recall in terms of offloading the task to fog network by using the harmonic mean.

(23) $$\begin{array}{c} \text{F }-\text{score }=\;2\frac{\text{recall x precision}}{\text{recall }+\text{ precision}}\\ \;=2\frac{2\text{ x TP}}{2\text{ x TP }+\text{ FN }+\text{ FP}}. \end{array}$$

Sensitivity (recall) is calculated as the number of tasks offloaded to fog correctly predicted divided by the total number of tasks offloaded to fog. In other words, the fraction of related tasks is retrieved.

(24) $$\begin{array}{c} \text{sensitivity }=\;\frac{\text{TP}}{\text{TP}+\text{FN}}. \end{array}$$

Specificity is calculated as the number of predictions for cloud data center correctly classified divided by all predictions for the cloud data center.

(25) $$\begin{array}{c} \text{specificity }=\;\frac{\text{TN}}{\text{TN}+\text{FP}}. \end{array}$$

Positive predictive value (precision) is the probability that a task offloaded to fog truly has to be offloaded to the fog network.

(26) $$\begin{array}{c} \text{PPV }=\;\frac{\text{TP}}{\text{TP}+\text{FP}}. \end{array}$$

Negative predictive value is the probability that a task offloaded to a cloud data center truly has to be offloaded to a cloud data center.

(27) $$\begin{array}{c} \text{NPV }=\;\frac{\text{TN}}{\text{FN}+\text{TN}}. \end{array}$$

**Figure 13:**
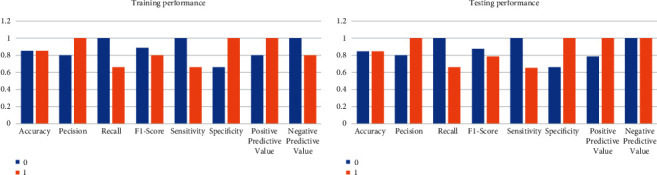
Performance measures (training and testing).

Various performance measures of both training and testing processes are described graphically in Figure 13 where both values (0 and 1) of label variables demonstrate their predictive rate. These performance measures give more meaning and build confidence where the consent of domain experts, according to the proposed algorithm, is essential to obtain the maximum benefits by the experiments as can be achieved. The prediction accuracy of 85.61% is obtained by the proposed algorithm; i.e., logistic regression with a high precision value for the fog network is 100% and 80.3% for the cloud data center. Likewise, sensitivity and specificity show 67.1% for the fog network and 100% for the cloud data center, respectively. The misclassification rate is 14.39%, the positive likelihood ratio is 2.89, and the negative likelihood ratio is 0.0. The support is the number of occurrences of each class in the test dataset for fog offloading which is 1322 and 1678 for the cloud data center.

**Figure 14:**
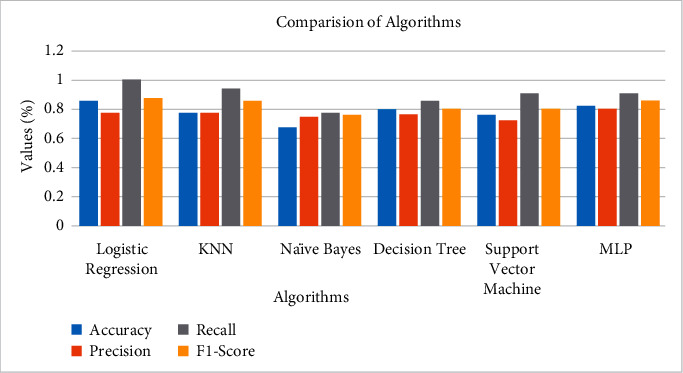
Comparisons of the proposed model with other algorithms.

Comparing the simulation results of the proposed model with other algorithms in Figure 14 demonstrates that the proposed algorithm gets the highest accuracy. The proposed model gets the highest accuracy reaching an 86% score and the lowest error/loss among others. On the contrary, Naïve Bayes could achieve only 68% accuracy which is the lowest. Comparative analysis using performance measures, i.e., precision, recall, and F1-score, among these algorithms is carried out on the basis of accuracy that measures how frequently the algorithms classify the data accurately.

**Figure 15:**
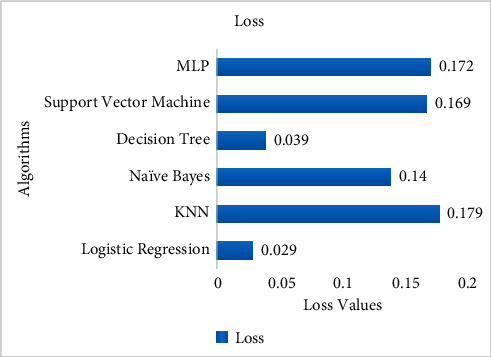
Error/loss comparison.

The proposed model exhibits the lowest loss, i.e., 0.029 as shown in Figure 15. The least loss value indicates how well the proposed model performs after every iteration of optimization. On the other hand, MLP infers 0.172 loss which is the highest among all other algorithms. Certainly, it is not always the case, but normally an increase in accuracy is observed with the decrease in loss accordingly.

**Figure 16:**
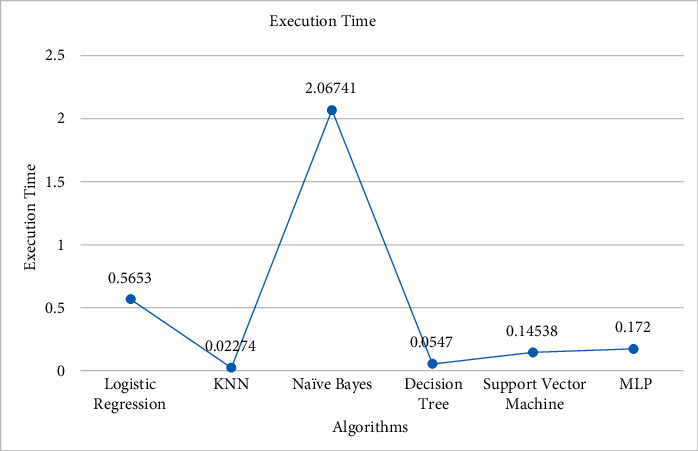
Execution time comparison.

However, while seeing the execution time of all algorithms in Figure 16 that are used in the simulation setup, the logistic regression model takes relatively more execution time to complete its operations. In view of the fact that the dataset contains few (10,000) instances to be processed by logistic regression for its training and testing purpose, on the other hand, thousands and even million records/instances can show the worth of the proposed model to perform its intended operations in an ideal short time where other classifiers may exhibit poor execution time. Naïve Bayes exhibits longest execution time, i.e., 2.06741.

**Figure 17:**
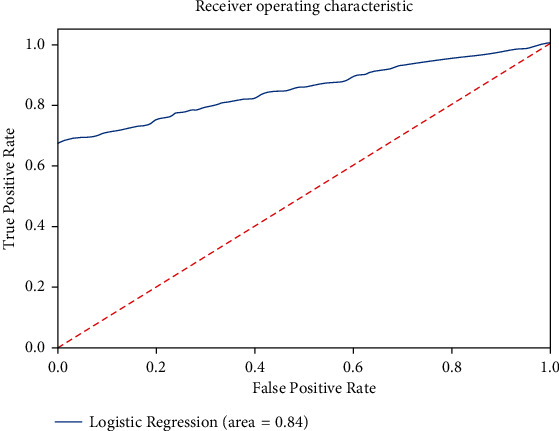
Receiver operating characteristics (ROC) curve performance.

The receiver operating characteristic (ROC) curve is another common tool used with binary classifiers. The dotted line represents the ROC curve of a purely random classifier. The ROC curve is used to identify the mutual association between the true negative (TN) rate and the false alarm rate in a communication channel in signal channel theory. It contains the capability to address the effectiveness and proficiency of a classification system that is binary in nature as the label variable possesses either fog or cloud characteristics. An efficient logistic regression training with a ROC curve is employed in Figure 17 where performance analysis is depicted. The ROC curve demonstrates the performance of the binary classification system as it forms with the true positive rate against the false-positive rate. It can be examined that the improved performance of logistic regression is reflected.

## 5. Conclusion

In this paper, a machine learning-based intelligent task offloading model is proposed in the fog-cloud collaborative network using a logistic regression algorithm. First, an offloading-related optimization problem is addressed by considering the threshold values of the concerned parameters associated with the cloud data center. Numerous types of applications including delay-sensitive and computation-intensive applications specifically need to perform their intended tasks as per their computing resource requirements that must be provisioned proportionately. Second, the projected model is proposed by employing an intelligent task offloading management system that predicts the incoming tasks generated by heterogeneous IoT and mobile devices that are located at some dispersed remote locations. Simulation results demonstrate that the proposed model is capable of predicting the task offloaded to either fog or cloud network successfully with 86% highest accuracy among other employed algorithms. Two cloud data centers located in America and Singapore and five fog nodes located at local geolocation with adequate hardware specification in terms of computation power, storage capacity, handling MIPS, upload and download bandwidth, and processor burst time have been employed in the whole simulation process. The mathematical model proves to validate the simulation process accordingly.

It is viable to mention that more work would be incorporated in the proposed model particularly in multifog offloading and distributed environment. What lacks in this model is first of all fog-to-fog communication where tasks possibly are offloaded to nearby fog computing network instead of routing to cloud. In order to strengthen the influence of fog computing, therefore, the second limitation is associated with the deployment of the proposed model in a distributed environment where data generated devices are located at some scattered locations. Furthermore, OMS needs to be more dynamic, so it can handle the intended tasks efficiently. Since services offered by fog computing are of large scale that usually raise trust issues and communication challenges, therefore, a prescribed strategy needs to be devised with the intention of handling these issues to reduce the impacts of the limitations of the proposed model.

In view of the fact that IoT devices generate such tasks that might be versatile and diverse in nature, therefore, it is not feasible that one should rely only on just data types generated by the mentioned devices but rather train the network; hence, the network performance significantly improves. The next research work will be based on updating the model in which the parameters, which are associated with the task offloading process, will be analyzed, improved, and proclaimed accordingly using the Adaptive Neuro-Fuzzy Inference System (ANFIS). ANFIS is an intelligent system that possesses the capability to be applied in such revolutionized areas where decision-making support is required; therefore, it would be recommended approach to continue the ongoing research work with the fog-cloud task offloading process.

## Figures and Tables

**Algorithm 1:**
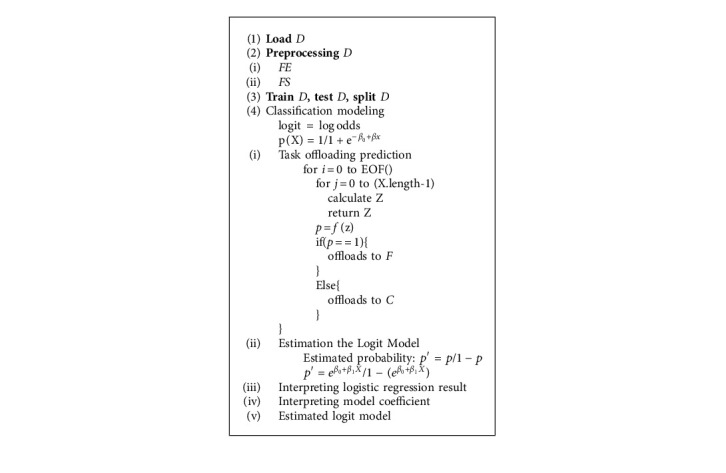
Task offloading in fog-cloud collaboration.

**Table 1:** Description of dataset attributes.

Sr	Attribute	Description	Type	Missing values
1	ID	Uniquely identified tuple identification number	Text	0
2	Size	Size of a tuple	Numeric	0
3	Name	Name of a tuple	Text	0
4	MIPS	Million instructions per second	Numeric	0
5	NumberOfPes	Number of processing elements	Numeric	0
6	RAM	How much memory is required	Numeric	0
7	BW	Bandwidth of a tuple required	Numeric	0
8	Source	Tuple originating source	Numeric	10,000
9	Destination	Tuple processing server/node	Numeric	10,000
10	Delay	Tuple delay details	Numeric	0
11	Priority	Urgency/importance of a tuple	Categorical	0
12	CloudletScheduler/PreviousTime	Scheduling information of a tuple	Numeric	0
13	CloudletScheduler/CurrentMips	Scheduling and sharing information	Numeric	10,000
14	CurrentAllocatedSize	Size of a tuple allocated at the time	Numeric	0
15	CurrentAllocatedRam	Amount of RAM allocated to a tuple	Numeric	0
16	CurrentAllocatedBw	Amount of bandwidth allocated	Numeric	0
17	CurrentAllocatedMips	Amount of MIPS allocated to a tuple	Numeric	0
18	BeingInstantiated	Status of a tuple	Categorical	0
19	GeoLocation/latitude	Latitude location of the tuple source	Numeric	0
20	GeoLocation/longitude	Longitude location of the tuple	Numeric	0
21	DataType	Data types of tuples, i.e., abrupt, bulk, large, location based, medical, etc.	Categorical	0
22	DataPercentage	Data size	Numeric	0
23	Tuple_Reversed	Tuple reversed from fog to cloud	Categorical	0
24	IsServerFound	If tuple found any server to be	Numeric	0
25	IsCloudServed	Is tuple served at cloud data center	Categorical	0
26	IsServed	Is tuple served by any server	Categorical	0
27	DeviceType	Actuators, dumb objects, mobile, node, sensor	Categorical	0
28	Service	Is tuple being served	Numeric	0
29	QueueDelay	If tuple finds delay while in queue	Numeric	0
30	InternalProcessingTime	Time taken to be processed	Numeric	0
31	FogLevelServed	If tuple is served at a fog node	Numeric	0
32	IsServedByFC_Cloud	If tuple is served at a cloud server	Numeric	0
33	BurstTime	Total burst time of a tuple	Numeric	0
34	BurstTimeDifference	Difference of burst time	Numeric	0
35	IsServedByFC (output)	Tuple serves at cloud server	Categorical	0

**Table 2:** Cloud data centers used in the setup.

DC	Geolocation	Memory (MB)	Storage (MB)	MIPS	BW (kbps)	Arch	OS	Status
USA data center	37.422421, −2.0866703	51200	1000000	500000	50000	x86	Linux	Live
Singapore data center	1.277911, 103.849662	51200	1000000	500000	50000	x86	Linux	Live

**Table 3:** Fog nodes used in the setup.

Sr	Name	Size	MIPS	RAM	UpBW	DownBW	Processor burst time
1	PakFog-0	25000	110000	16384	2500	1700	25
2	PakFog-1	10000	50000	6144	1000	700	25
3	PakFog-2	20000	95000	12288	2000	1500	25
4	PakFog-3	15000	85000	10240	1500	1200	15
5	PakFog-4	12000	75000	8192	1200	1000	30

**Table 4:** Symbol description.

Symbol	Definition
*D*	Dataset
FE	Feature engineering
FS	Feature selection
Logit	Logistic unit (log odds)
*p* (*X*)	Sigmoid function
EOF()	End of file
*Z*	Weighted sum
*β*_0_	Intercept or bias term
*β*_1_	Coefficient
*X*	Features
*F* (*z*)	Calculate probability
*C*	Cloud data center
*F*	Fog network
*p*′	Predicted/Estimated probability

**Table 5:** Statistical measures of the data.

	Size	MIPS	RAM	BW	Geo/latitude	Geo/longitude	BurstTime	IsServedByFC
Count	10000	10000	10000	10000	10000	10000	10000	10000
Mean	206.683	344.65	418.43	88.494	33.68742	73.0078	12.662	0.4326
std	74.722	350.36	325.31	39.0848	0.065175	0.084029	9.305505	0.495461
min	80	50	100	20	33.57106	72.83865	0	0
25%	170	100	200	80	33.63828	72.94511	10	0
50%	220	200	300	90	33.70275	73.01083	10	0
75%	270	500	500	100	33.73518	73.09606	15	1
max	300	1200	1024	150	33.78799	73.14154	35	1

## Data Availability

The data used in this paper can be requested from the corresponding author upon request.
